# Supplementary material for: Transcriptome dynamics of the Myxococcus xanthus multicellular developmental program
Source: eLife. 2019 Oct 14;8:e50374. doi: 10.7554/eLife.50374 (PMC6791715; doi:10.7554/eLife.50374)
Supplement: Transparent reporting form [file elife-50374-transrepform.docx]

***eLife’s* transparent reporting form**

We encourage authors to provide detailed information *within their submission* to facilitate the interpretation and replication of experiments. Authors can upload supporting documentation to indicate the use of appropriate reporting guidelines for health-related research (see [EQUATOR Network](http://www.equator-network.org/%20)), life science research (see the [BioSharing Information Resource](https://biosharing.org/)), or the [ARRIVE guidelines](http://www.plosbiology.org/article/info:doi/10.1371/journal.pbio.1000412) for reporting work involving animal research. Where applicable, authors should refer to any relevant reporting standards documents in this form.

If you have any questions, please consult our Journal Policies and/or contact us: [editorial@elifesciences.org](mailto:editorial@elifesciences.org).

**Sample-size estimation**

- You should state whether an appropriate sample size was computed when the study was being designed
- You should state the statistical method of sample size computation and any required assumptions
- If no explicit power analysis was used, you should describe how you decided what sample (replicate) size (number) to use

Please outline where this information can be found within the submission (e.g., sections or figure legends), or explain why this information doesn’t apply to your submission:

We have indicated in the section of Material and Methods entitled “Preparation of cells for RNA-Seq experiment” the number of plates harvested at each time point. Moreover, we have indicated the number of sequences obtained in section “Transcriptome analysis of the developmental program by RNA-Seq” of Results and Discussion.

**Replicates**

- You should report how often each experiment was performed
- You should include a definition of biological versus technical replication
- The data obtained should be provided and sufficient information should be provided to indicate the number of independent biological and/or technical replicates
- If you encountered any outliers, you should describe how these were handled
- Criteria for exclusion/inclusion of data should be clearly stated
- High-throughput sequence data should be uploaded before submission, with a private link for reviewers provided (these are available from both GEO and ArrayExpress)

Please outline where this information can be found within the submission (e.g., sections or figure legends), or explain why this information doesn’t apply to your submission:

Two biological replicates were analyzed in this study by RNA-Seq, and this information is found in several sections along the manuscript, but especially in section “Transcriptome analysis of the developmental program by RNA-Seq” of Results and Discussion and section “Preparation of cells for RNA-Seq experiment” of Material and Methods. Criteria to include a gene within any of the 10 developmental groups are also indicated in the section “Gene expression profiles organize into 10 developmental groups (DGs)” and the section “Developmental gene and analysis” of Material and Methods. In the case of beta-galactosidase activity, three biological replicates were used, and this information is stated in the section “Assay of β-galactosidase activity” of Material and Methods.

**Statistical reporting**

- Statistical analysis methods should be described and justified
- Raw data should be presented in figures whenever informative to do so (typically when N per group is less than 10)
- For each experiment, you should identify the statistical tests used, exact values of N, definitions of center, methods of multiple test correction, and dispersion and precision measures (e.g., mean, median, SD, SEM, confidence intervals; and, for the major substantive results, a measure of effect size (e.g., Pearson's r, Cohen's d)
- Report exact p-values wherever possible alongside the summary statistics and 95% confidence intervals. These should be reported for all key questions and not only when the p-value is less than 0.05.

Please outline where this information can be found within the submission (e.g., sections or figure legends), or explain why this information doesn’t apply to your submission:

The statistical methods used are described in Material and Methods. Sections “Sequencing and transcriptomic data analysis” and “Developmental gene and analysis”.

(For large datasets, or papers with a very large number of statistical tests, you may upload a single table file with tests, Ns, etc., with reference to sections in the manuscript.)

**Group allocation**

- Indicate how samples were allocated into experimental groups (in the case of clinical studies, please specify allocation to treatment method); if randomization was used, please also state if restricted randomization was applied
- Indicate if masking was used during group allocation, data collection and/or data analysis

Please outline where this information can be found within the submission (e.g., sections or figure legends), or explain why this information doesn’t apply to your submission:

I think group allocation does not apply to our submission. However, we have indicated that to analyze development we collected samples at 0, 6, 12, 24, 48, 72 and 96 h after starvation (section “Preparation of cells for RNA-Seq experiment” of Material and Methods and most figures and results.

**Additional data files (“source data”)**

- We encourage you to upload relevant additional data files, such as numerical data that are represented as a graph in a figure, or as a summary table
- Where provided, these should be in the most useful format, and they can be uploaded as “Source data” files linked to a main figure or table
- Include model definition files including the full list of parameters used
- Include code used for data analysis (e.g., R, MatLab)
- Avoid stating that data files are “available upon request”

Please indicate the figures or tables for which source data files have been provided:

Two source data documents in Excel format are provided for Table 1.

One source data document in Excel format is provided for Figure 2.

Seven source data documents in Excel format are provided for Figure 3.

One source data document in Excel format is provided for Figure 11.
